# Supplementary material for: Novel prokaryotic system employing previously unknown nucleic acids-based receptors
Source: Microb Cell Fact. 2022 Oct 4;21:202. doi: 10.1186/s12934-022-01923-0 (PMC9531389; doi:10.1186/s12934-022-01923-0)
Supplement: Supplementary file 1 — Additional file 1: Table S1. Effect of primary TezR–D1/R1 removal on bacterial size [file 12934_2022_1923_MOESM1_ESM.docx]

Tetz V. Tetz G. Novel prokaryotic system employing previously unknown nucleic acids-based receptors.

Supplementary table 1. Effect of primary TezR–D1/R1 removal on bacterial size.

| Bacteria | Bacterial size (px2) | SD | p |
| --- | --- | --- | --- |
| Control *S. aureus* | 52.3 | 2.08 |  |
| *S. aureus* TezR–D1^d^/R1^d^ | 59.1 | 6.94 | <0.001 |
| Control *E. coli* | 40.1 | 3.62 |  |
| *E. coli* TezR–D1^d^/R1^d^ | 42.2 | 5.77 | 0.055 |
