# Supplementary material for: Novel prokaryotic system employing previously unknown nucleic acids-based receptors
Source: Microb Cell Fact. 2022 Oct 4;21:202. doi: 10.1186/s12934-022-01923-0 (PMC9531389; doi:10.1186/s12934-022-01923-0)
Supplement: Supplementary file 2 — Additional file 2: Table S2. Effect of primary TezRs removal on the size of B. pumilus VT1200 biofilm [file 12934_2022_1923_MOESM2_ESM.docx]

Tetz V. Tetz G. Novel prokaryotic system employing previously unknown nucleic acids-based receptors.

Supplementary table 2. Effect of primary TezRs removal on the size of *B. pumilus* VT1200 biofilm.

| Bacteria | Biofilm size | SD | p |
| --- | --- | --- | --- |
| Control | 109290.7 | 17343.48 |  |
| TezR–D1^d^ | 158213.3 | 18154.98 | 0.028 |
| TezR–R1^d^ | 138990 | 6451.97 | 0.083 |
| TezR–D1^d^/R1^d^ | 103975.67 | 11843.53 | 0.687 |
| TezR–D2^d^ | 1076281 | 64089.26 | <0.001 |
| TezR–R2^d^ | 137660 | 17984.35 | 0.121 |
| TezR–D2^d^/R2^d^ | 288876.3 | 19735.85 | <0.001 |
| TezR–D1^d^/D2^d^ | 256159.6 | 32023.40 | 0.006 |
| TezR–D1^d^/R1^d^/D2^d^/R2^d^ | 134314 | 15963.79 | 0.14 |
