# Supplementary material for: Novel prokaryotic system employing previously unknown nucleic acids-based receptors
Source: Microb Cell Fact. 2022 Oct 4;21:202. doi: 10.1186/s12934-022-01923-0 (PMC9531389; doi:10.1186/s12934-022-01923-0)
Supplement: Supplementary file 3 — Additional file 3: Table S3. Effect of TezR removal on sporulation under normal conditions [file 12934_2022_1923_MOESM3_ESM.docx]

Tetz V. Tetz G. Novel prokaryotic system employing previously unknown nucleic acids-based receptors.

Supplementary table 3. Effect of TezR removal on sporulation under normal conditions.

| Bacteria | Sporulation (%) | SD | p |
| --- | --- | --- | --- |
| Control | 17.67 | 2.62 |  |
| TezR–D1^d^ | 76.33 | 5.312 | <0.001 |
| TezR–R1^d^ | 82 | 6.16 | <0.001 |
| TezR–D1^d^/R1^d^ | 21.67 | 2.05 | 0.11 |
| TezR–D2^d^ | 0 | 0 | 0.007 |
| TezR–R2^d^ | 96 | 4.32 | <0.001 |
| TezR–D1^d^/R1^d^/D2^d^/R2^d^ | 13.67 | 1.89 | 0.105 |
