# Supplementary material for: Novel prokaryotic system employing previously unknown nucleic acids-based receptors
Source: Microb Cell Fact. 2022 Oct 4;21:202. doi: 10.1186/s12934-022-01923-0 (PMC9531389; doi:10.1186/s12934-022-01923-0)
Supplement: Supplementary file 4 — Additional file 4: Table S4. Effect of TezR removal on sporulation under stress conditions [file 12934_2022_1923_MOESM4_ESM.docx]

Tetz V. Tetz G. Novel prokaryotic system employing previously unknown nucleic acids-based receptors.

Supplementary table 4. Effect of TezR removal on sporulation under stress conditions.

| Bacteria | Sporulation (%) | SD | p |
| --- | --- | --- | --- |
| Control normal conditions | 17.67 | 2.62 |  |
| Control stress conditions | 77.67 | 6.18 |  |
| TezR–D1^d^ | 21.67 | 3.40 | <0.001 |
| TezR–R1^d^ | 95.67 | 3.30 | 0.02 |
| TezR–D1^d^/R1^d^ | 92.33 | 3.40 | 0.035 |
| TezR–D2^d^ | 4 | 2.16 | <0.001 |
| TezR–R2^d^ | 97.67 | 2.05 | 0.022 |
| TezR–D1^d^/R1^d^/D2^d^/R2^d^ | 96.33 | 2.36 | 0.023 |
