# Supplementary material for: Novel prokaryotic system employing previously unknown nucleic acids-based receptors
Source: Microb Cell Fact. 2022 Oct 4;21:202. doi: 10.1186/s12934-022-01923-0 (PMC9531389; doi:10.1186/s12934-022-01923-0)
Supplement: Supplementary file 5 — Additional file 5: Table S5. MICs of tested reverse transcriptase inhibitors and integrase inhibitor against control S. aureus [file 12934_2022_1923_MOESM5_ESM.docx]

Tetz V. Tetz G. Novel prokaryotic system employing previously unknown nucleic acids-based receptors.

Supplementary table 5. MICs of tested reverse transcriptase inhibitors and integrase inhibitor against control *S. aureus*.

| **Drug** | **Concentration of drug in µg/mL** | | | | | | | | | | | | |
| --- | --- | --- | --- | --- | --- | --- | --- | --- | --- | --- | --- | --- | --- |
|  | **512** | **256** | **128** | **64** | **32** | **16** | **8** | **4** | **2** | **1** | **0.5** | **0.25** | **0.125** |
| Etravirine | +* | + | + | + | + | + | + | + | + | + | + | + | + |
| Nevirapine | + | + | + | + | + | + | + | + | + | + | + | + | + |
| Raltegravir | + | + | + | + | + | + | + | + | + | + | + | + | + |

* “+” was used to mark the presence of bacterial growth
