# Supplementary material for: Novel prokaryotic system employing previously unknown nucleic acids-based receptors
Source: Microb Cell Fact. 2022 Oct 4;21:202. doi: 10.1186/s12934-022-01923-0 (PMC9531389; doi:10.1186/s12934-022-01923-0)
Supplement: Supplementary file 6 — Additional file 6: Figure S1. Absence of RNase A internalization in B. pumilus [file 12934_2022_1923_MOESM6_ESM.pdf]

Tetz V. Tetz G. Novel prokaryotic system employing previously unknown nucleic acids-based receptors.

Supplementary Figure S1. Absence of RNase A internalization in *B. pumilus*.

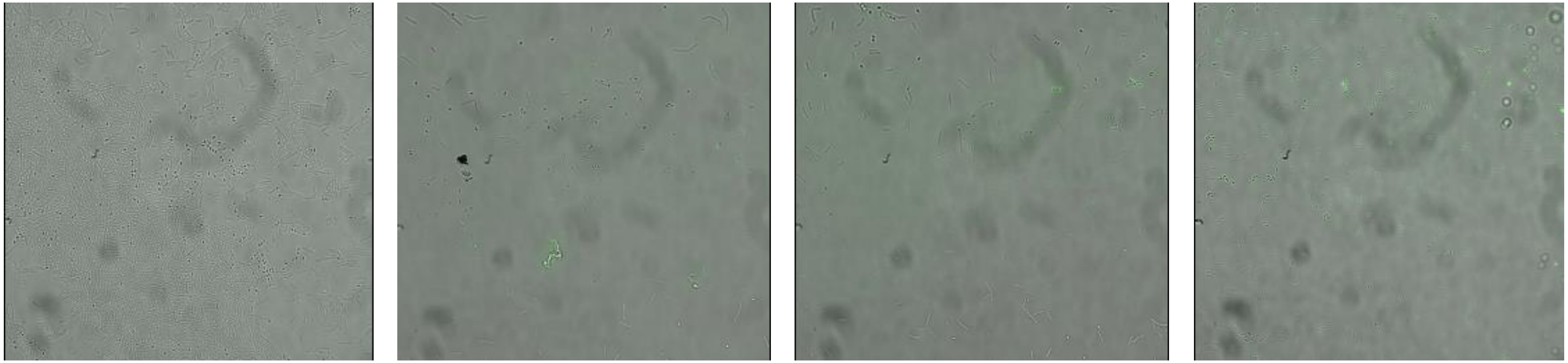

A B C D

B. pumilus cells were (A) untreated control (B) treated with fluorophore-labeled RNase A (100 µg/mL) for 15 min (C) treated with fluorophore-labeled RNase A (100 µg/mL) for 60 min (D) cultivated for 24h on agar supplemented with fluorophore-labeled RNase A (100 µg/mL)for 24h supplemented with labeled RNase A (100 µg/mL).
