# Supplementary material for: Novel prokaryotic system employing previously unknown nucleic acids-based receptors
Source: Microb Cell Fact. 2022 Oct 4;21:202. doi: 10.1186/s12934-022-01923-0 (PMC9531389; doi:10.1186/s12934-022-01923-0)

Tetz V. Tetz G. Novel prokaryotic system employing previously unknown nucleic acids-based receptors.

Supplementary Figure 2. Effect of TezRs removal on light sensing.

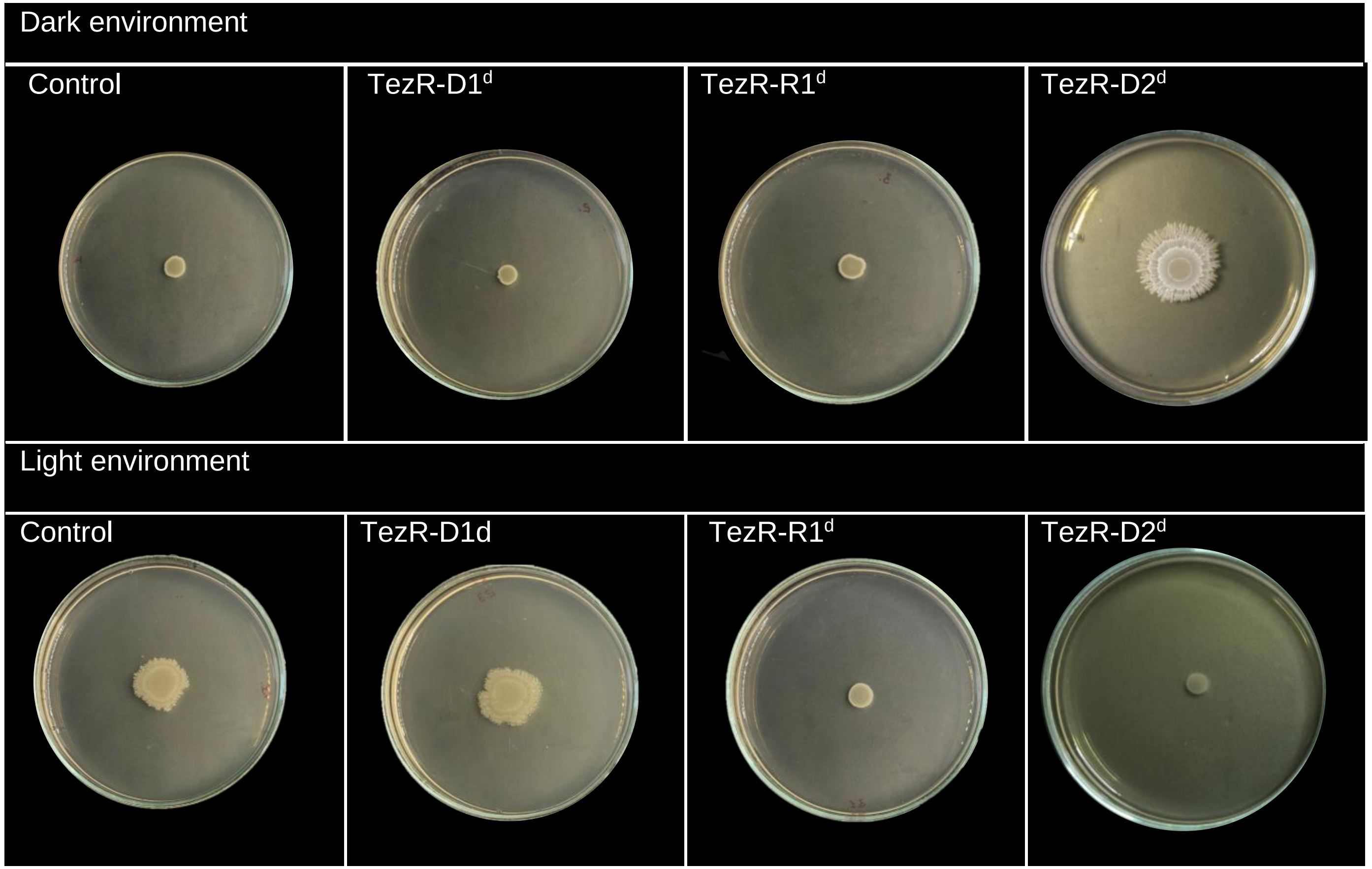

Supplement: Supplementary file 7 — Additional file 7: Figure S2. Effect of TezRs removal on light sensing [file 12934_2022_1923_MOESM7_ESM.pdf]
